# Out-Patients and Solicitors' Touts

**Published:** 1912-04-20

**Authors:** Reginald R. Garratt

**Affiliations:** Secretary. Royal Free Hospital, Gray's Inn Road, W.C.


### OUT-PATIENTS AND SOLICITORS' TOUTS.

*To the Editor of* The Hospital.

Sir,—On page 33 of your issue of the 13th inst., you refer to a paragraph recently appearing in *Truth* in regard to out-patients and solicitors' touts, and as the name of the Royal Free Hospital is mentioned I think it well to tell you that the paragraph has already been noted.

It is unfortunate that the office of "Legal Aid Society" is situated exactly opposite the gate of this Hospital. It is impossible to prevent persons connected with that Society noticing cases of accidents which may be admitted here, but in no case is any unauthorised person or even a solicitor allowed to enter any department of the hospital. The information that these societies obtain can only be through persons after leaving the hospital, and whom they may have observed to be connected with patients brought in.—I am, yours truly,

Reginald R. Garratt,
Secretary.
Royal Free Hospital,
Gray's Inn Road, W.C., April 13.

